# D-karyo—A New Prenatal Rapid Screening Test Detecting Submicroscopic CNVs and Mosaicism

**DOI:** 10.3390/diagnostics11020337

**Published:** 2021-02-18

**Authors:** Osamu Shimokawa, Masayoshi Takeda, Hiroyasu Ohashi, Akemi Shono-Ota, Mami Kumagai, Risa Matsushika, Chika Masuda, Kohtaro Uenishi, Ritsuko Kimata Pooh

**Affiliations:** Clinical Laboratory, Ritz Medical Co., Ltd., 1-24, Uehommachi, 7, Tennoji, Osaka 543-0001, Japan; shimokawa.osamu@ritz-med.com (O.S.); takeda.masayoshi@ritz-med.com (M.T.); ohashi.hiroyasu@ritz-med.com (H.O.); oota.akemi@ritz-med.com (A.S.-O.); kumagai.mami@ritz-med.com (M.K.); matsushika.risa@ritz-med.com (R.M.); masuda.chika@ritz-med.com (C.M.); uenishi.koutarou@fetal-medicine-pooh.com (K.U.)

**Keywords:** prenatal diagnosis, chromosome, karyotyping, digital, copy number variation, CNV, preimplantation genetic testing, PGT, next-generation sequencing, NGS, eXome Hidden Markov Model, XHMM, submicroscopic abnormality, mosaicism

## Abstract

Chromosomal microarray analysis (CMA), recently introduced following conventional cytogenetic technology, can detect submicroscopic copy-number variations (CNVs) in cases previously diagnosed as “cytogenetically benign”. At present, rapid and accurate chromosomal analysis is required in prenatal diagnostics, but prenatal CMA is not widely used due to its high price and long turnaround time. We introduced a new prenatal screening method named digital karyotyping (D-karyo), which utilizes a preimplantation genetic test for the aneuploidy (PGT-A) platform. First, we conducted a preliminary experiment to compare the original PGT-A method to our modified method. Based on the preliminary results, we decided to implement the modified strategy without whole-genome amplification (WGA) and combined it with three analytical software packages. Next, we conducted a prospective study with 824 samples. According to the indication for invasive tests, the D-karyo positive rates were 2.5% and 5.0%, respectively, in the screening positive group with NT ≥ 3.5 mm and the group with fetal abnormalities by ultrasound. D-karyo is a breakthrough modality that can detect submicroscopic CNVs ≥ 1.0 Mb accurately in only 10.5 h for 24 samples at a low cost. Implementing D-karyo as a prenatal rapid screening test will reduce unnecessary CMA and achieve more accurate prenatal genetic testing than G-banding.

## 1. Introduction

Conventional cytogenetic karyotyping is performed by G-banding. This analog karyotyping has several disadvantages compared to recent molecular genetic approaches. G-banding requires a long duration (10–14 days) for cell culture. Prenatal karyotyping has lower band resolution than postnatal specimen testing [1,2], and can occasionally overlook small structural aberrations. Furthermore, because microscopic observation requires a high degree of specialization, it is highly dependent on the examiner’s expertise and experience. In addition, regarding mosaicism, the mosaic frequency may change during cell culture because the cells with high proliferative capacity can divide and multiply much more than cells with low capacity. Recent molecular genetic approaches, including chromosomal microarray analysis (CMA), have been considered new tools to compensate for traditional karyotyping disadvantages. CMA has been broadly used for prenatal genetic testing in the United States and European countries. Numerous reports have been published about the detection rates for pathogenic copy-number variations (CNVs) in fetuses with structural anomalies, detected by ultrasound scanning using CMA, for postnatal diagnosis and prenatal settings [3,4,5,6]. Depending on the type of anomaly groups, the detection rates of pathogenic CNVs are different. The rate was found to be 6.0% in abnormalities using ultrasonography [3], 9.2% in cardiac anomalies, which contain pathogenic and likely pathogenic CNVs [4], 17.0% in anomalies by prenatal ultrasound screening [5], and 9.2% in cardiac abnormalities, suggesting approximately 10% on average. By comparison, the pathogenic CNV rate in advanced maternal age was reported to be as low as 1.7% [3]. The American College of Obstetricians and Gynecologists (ACOG) and the Society for Maternal-Fetal Medicine (SMFM) provided the joint recommendation in 2016 that CMA is performed as a prenatal diagnosis for a fetus that has one or more structural anomalies detected by ultrasonography [7]. The UK health service stated that CMA should replace karyotyping from the viewpoint of the detection rate and cost-effectiveness [8].

According to the Japanese survey for prenatal genetic testing in 2016, 35,900 cases of maternal serum screening, 18,600 cases of amniocentesis (AC), 13,600 cases of non-invasive prenatal testing (NIPT), and 1950 cases of chorionic villus sampling (CVS) were reported [9]. The invasive tests were mostly conducted with conventional karyotyping, and there was no description of CMA. The CMA has not been well utilized in Japan because of expensive CMA-related reagents, the long turnaround time (TAT), and low-throughput modality. 

In our laboratory, we perform cytogenetic and molecular-genetic tests in more than 2000 prenatal samples per year. In all prenatal samples, we perform quantitative fluorescent polymerase chain reaction (QF-PCR) to detect major trisomies and sex chromosome aneuploidy (SCA), and G-banding for karyotyping. As we experienced a few cases in which a prenatal normal karyotype was diagnosed as microdeletion syndrome after birth, there was a dilemma in the detection limit of G-banding.

Recently, assisted reproductive technology has advanced remarkably. In the clinical practice of In Vitro Fertilization-Embryo Transfer (IVF-ET), the preimplantation genetic testing for aneuploidy (PGT-A) has been widely used to validate chromosomal aneuploidies of IVF eggs before ET. Various techniques have previously been developed and applied for PGT-A [10]. Initially, fluorescent in situ hybridization (FISH) with targeting probes for imbalanced chromosomal anomalies derived from carrier parents with Robertsonian and balanced translocation has made it possible to detect limited numbers of chromosomal aneuploidies [11]. Subsequently, analysis for quantitative polymerase chain reaction (qPCR) [12,13] emerged. Furthermore, single nucleotide polymerase (SNP) array [14,15] and array comparative genome hybridization (aCGH) [16,17,18,19] have gradually become the primary techniques for comprehensive chromosome analysis. Most recently, the next-generation sequencing (NGS)-based technique has been applied for PGT-A [20,21,22].

After considering the current situation objectively with limitations of G-band, the cost issue of CMA, and development of PGT-A using NGS, an additional approach was conceived. By applying PGT-A technology, combined with G-banding for prenatal specimens of CVS and AC, we assumed that prenatal genetic testing might be performed rapidly and accurately, with minimal reductions in inter-examiner errors. Currently, the Illumina VeriSeq^TM^ PGS high-throughput system allows us to examine at least 24 samples simultaneously at a resolution of ≥10 Mb, which is equivalent to conventional karyotyping [23,24]. A previous study shows the minimum detectable size on CNV using the VeriSeq^TM^ PGS is assumed to be approximately 10 Mb [25], and some variations, such as 14 Mb, have also been reported [21]. Furthermore, it was reported that more than 20% frequency of mosaicism would be detected by the subsequent analysis with Illumina BlueFuse Multi software [26]. Recent array-based testing for chromosomal aneuploidies, i.e., CMA, has also been developed to provide accurate information, including regional loci, sizes, and more than 20% of mosaicisms CNVs in the highest resolution (cut-off for losses; 50 kb). However, the CMA system with such a high resolution would allow the examination of a limited number of samples simultaneously and would be time consuming. 

This article introduces a new cost-effective and time-saving prenatal screening method named digital karyotyping, hereafter D-karyo. D-karyo utilizes the platform of PGT-A technology. However, when the PGT-A platform is used with the original protocol, the resolution may be only slightly superior or similar to that of the G-banding. Therefore, we decided to increase the CNV detection resolution using two measures. First, in this study, sufficient DNA could be obtained from prenatal specimens, so we examined whether the CNV detection rate could be improved by omitting whole-genome amplification (WGA). Second, we simultaneously introduced three different analytical tools for statistical analyses of NGS data for better resolution and mosaic detection. The statistical analytical tools used in this study were the eXome Hidden Markov Model (XHMM) [27], the quantitative DNA sequencing for chromosomal aberrations (QDNAseq) [28], and a comparative analytical method. 

The first purpose of this study was to verify how small CNVs can be reliably detected by the original PGT method and our modified methods to find the detection limit and detection rate using known pathogenic CNVs, and to determine the most practical and reliable method for the subsequent prospective study. The second purpose was to confirm the usefulness of the D-karyo implementation as a rapid screening test combined with G-banding, by testing 824 clinical samples with the best method in the initial study.

## 2. Materials and Methods

First, we conducted an experimental verification study to confirm the accuracy of D-karyo. We verified the detection limit and rate of different D-karyo methods. We used three methods and examined 21 known microdeletions/microduplications already confirmed by SNP microarray. Initially, PGT-A strongly requires whole-genome amplification (WGA) due to an insufficient DNA amount because PGT-A is a genetic test of 5–6 cells biopsied from the trophectoderm. In the original PGT-A protocol with Illumina BlueFuse Multi software, WGA is mandatory. As it was reported that the detection limit of BlueFuse Multi was 10 Mb CNVs, as described above, we utilized other statistical software, namely, XHMM [27], which can detect smaller CNVs. We also considered that WGA may not always be necessary because a sufficient amount of gDNA was obtained from the prenatal sample. Therefore, we compared the detection limit and detection rate among three methods: the original PGT method, the modified method with WGA, and the modified method without (w/o) WGA. 

Next, based on the results of the initial validation study, we conducted a D-karyo clinical trial in actual clinical cases using XHMM w/o WGA. Although XHMM is the software that detects CNVs, it was predicted that many mosaic cases would be included in actual cases, so we introduced the additional two analytic tools to detect mosaics. A total of 913 invasive prenatal specimens were collected at the Fetal Diagnostic Center, CRIFM Clinical Research Institute of Fetal Medicine in Osaka, Japan, for nine months between March and November of 2020. All mothers underwent prior ultrasonography to detect fetal abnormalities and received genetic counseling. They decided to undergo an invasive test after genetic counseling. The main indications for invasive tests were positive Down’s syndrome screening, fetal anomalies detected by ultrasonography, advanced maternal age, and others including family history, previous pregnancy with chromosomal abnormalities, and parental anxiety. All participants provided written, informed consent, approved by the Institutional Review Boards (No. CRI-IRB-014). Inclusion criteria were singleton pregnancy at 11 or more weeks of gestation, with maternal age over 18. Exclusion criteria were twin or higher-order pregnancies. D-karyo was performed on 824 samples, including 749 CVSs and 75 ACs, after excluding 89 cases with trisomy of chromosome 13, 18, and 21, and SCA detected by QF-PCR. 

### 2.1. Molecular Cytogenetic Analysis

Genomic DNA (gDNA) was directly extracted using DNeasy^®^ Blood & Tissue Kit (Qiagen, Hilden, Germany), from 5–10 mg of chorionic villi separated from decidua under a microscope, or 10 ml of amniotic fluid. The gDNA was divided into a portion for QF-PCR and a portion for subsequent tests. QF-PCR was performed first to detect major trisomies and sex chromosome aneuploidy under the manufacturer’s protocol using AnueFast^TM^ QF-PCR Kit (Genomed Ltd., Kent, UK), and the remaining gDNA was used in D-karyo. The samples that remained after extracting the DNA were cultured and subjected to G-banding. Karyotyping was conducted following the conventional standard protocol.

The gDNA source is different according to the distinct differentiation step of early cell lineage. That is, the target of each examination is different. D-karyo deals with uncultured chorionic villi, which contains trophoblast and mesenchyme. G-banding of CVS deals with long-term cultured chorionic villi, which represent the mesenchymal core of the villus. G-banding of AC deals with cultured amniotic fluid cells, containing origins from the epiblast of the inner cell mass. Therefore, it is expected that the outcomes in each method reflect each gDNA source. 

Confined placental mosaicisms (CPM) for type I, II, III, and true fetal mosaicism (TFM) were classified by following the standard method [29]. In cases with mosaicism, uncultured FISH on interphase nuclei was conducted to verify the accurate mosaic rate, using AneuVysion Multicolor DNA Probe Kit (Vysis CEP 18/X/Y—alpha satellite/LSI 13/21) (Abbott Molecular Inc, Des Plaines, IL, USA). CNVs and their accurate sizes were assessed by SNP microarray with CytoScan^TM^ HD or 750K Array (Affymetrix, Santa Clara, CA, USA) following the manufacturer’s instructions. Subsequent data analysis was performed with Chromosome Analysis Suite (ChAS) (Affymetrix, Santa Clara, CA, USA) and the output data was compared with the two databases of the Database of Genomic Variants (DGV; http://dgv.tcag.ca/dgv/app/home (accessed on 1 December 2020)) and DECIPHER (https://decipher.sanger.ac.uk (accessed on 1 December 2020)). Discrepancies between G-banding and D-karyo were additionally investigated by SNP microarray or FISH method.

### 2.2. Next-Generation Sequencing (NGS) and Data Analysis

From the initial study result, we performed D-karyo w/o WGA. According to the manufacturer’s instructions using VeriSeq^TM^ PGS Kit (Illumina Inc., San Diego, CA, USA), we prepared NGS libraries. Samples w/o WGA were directly proceeded to VeriSeq Library preparation by skipping the step of SurePlex^TM^ DNA Amplification System (Illumina Inc., San Diego, CA, USA). Final pooled libraries were sequenced with dual index single-end 36 base pair reads on the Illumina MiSeq^®^ platform (Illumina Inc., San Diego, CA, USA). We performed the data analysis, including demultiplexes and alignments of reads to the human reference genome (GRCh37), on MiSeq Reporter Software (Illumina Inc., San Diego, CA, USA). Subsequently, we analyzed chromosomal aneuploidies by BlueFuse Multi v4.5 Software (Illumina Inc., San Diego, CA, USA). We performed integrated copy-number detection with the following steps. First, raw sequencing data (fastq) were aligned to the human reference genome (human_g1k_v37_decoy.fasta) by Burrows-Wheeler Aligner (BWA) v0.7.17. Subsequently, the potential PCR duplications were removed by SAMTOOLS (generation of BAM files) and reads with mapping quality <20 were excluded. Then, we counted the final numbers of reads. To evaluate CNVs, the Z-score was calculated with the eXome Hidden Markov Model v1.0 (XHMM), which was originally developed to detect CNVs from targeted exome sequencing data [27]. In our analysis, we adjusted the length of bins depending on the ability of sequences to be mapped to reduce false deletion events [30]. We used 100 in-house controls of 50 males and 50 females.

CNVs were detected by both XHMM automatic detection system and the additional standard for CNV detection, i.e., three consecutive target regions with Z-score of 2.5 or more.

CNVs on samples w/o WGA were also analyzed by quantitative DNA sequencing (QDNAseq), a tool to quantify chromosomal copy-number aberrations [28] from BAM files extracted in the same manner as above, for which analysis was performed by 1 Mb-bin. The consequent result was expressed as the Log2 ratio, which enabled addressing the mosaic aneuploidy of the whole chromosome. QDNAseq can reflect the mosaic rate as a percentage but may overlook low-frequency mosaics. To compensate for this weak point of QDNAseq, we added another comparative analysis, evaluating the target chromosome’s aneuploidy by quantifying how much the target fragment amount changed compared with the normal reference group using the Z-score, calculated from the mean of 100 in-house controls. The comparative analysis is useful for detecting low-frequency mosaic rates of 10–20%. Therefore, we evaluated mosaicism by both Log2 ratio from QDNAseq and Z-score from comparative analysis.

### 2.3. Data Interpretation on Pathogenicity of CNVs

CNVs, which contained genes in the online mendelian inheritance in Man (OMIM; https://www.ncbi.nlm.nih.gov/omim (accessed on 1 December 2020)), resulting in duplications ≥ 2.0 Mb and deletions ≥ 1.0 Mb, were flagged whereas CNVs in DGV were not flagged. Any small sizes of CNVs, which were possible to become pathogenic or likely to be pathogenic, were reported by referred to DECIPHER and ClinGen (https://dosage.clinicalgenome.org/pathogenic_region.shtml (accessed on 1 December 2020)). Detected CNVs were classified into five categories as “pathogenic”, “likely pathogenic”, “variant of uncertain significance (VUS)”, “likely benign”, and “benign” by scoring according to the American College of Medical Genetics (ACMG) and Genomics Guidelines 2020 for the interpretation and reporting of CNVs [31]. 

### 2.4. Statistical Analysis

Filtered mapped reads as a percentage of total numbers of reads regarding data with and w/o WGA and averages of Z-scores on gains and losses in XHMM were evaluated with simple linear regression analysis. *p* < 0.05 was considered statistically significant. Statistical analyses were performed with R version 4.0.3.

## 3. Results

### 3.1. Results of the First Verification Experiment Comparing Three Methods of D-karyo

**Figure 1:**
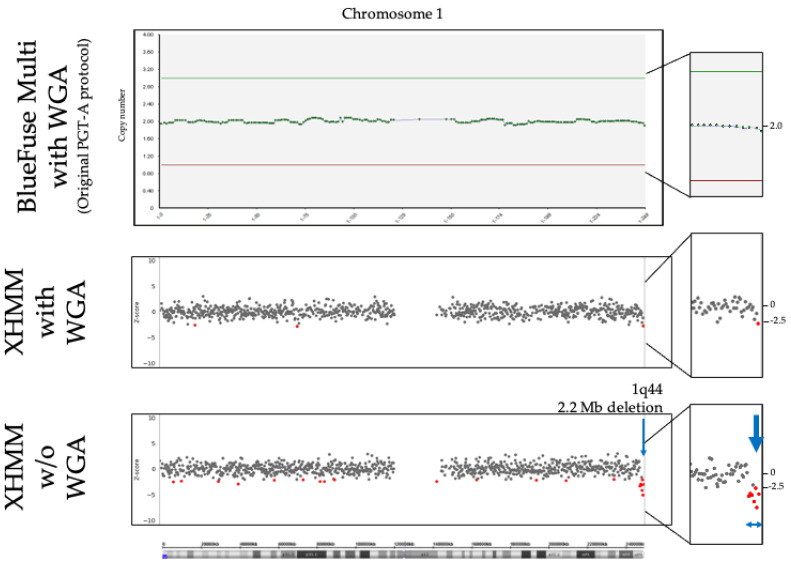
Comparison of actual data obtained from three D-karyo methods; BlueFuse Multi with whole-genome amplification (WGA), eXome Hidden Markov Model (XHMM) with WGA, and XHMM w/o WGA. 1q terminal 2.2 Mb deletion in Case pre-6 was detected by only the XHMM w/o WGA method (Lower bar). Blue arrows indicate the terminal deletion, with multiple red dots. BlueFuse Multi (upper bar) cannot detect CNVs, and XHMM with WGA (middle bar) shows only one red dot, which was interpreted as normal.

In the initial validation study, we verified the detection limit and detection rate of each D-karyo method. Focusing on the minimum size of CNVs in this preliminary study, BlueFuse Multi with WGA (original PGT-A method) detected 5.3 Mb or larger CNVs. XHMM with WGA detected 855 kb CNVs but missed 2.2 Mb terminal deletion, 50% mosaicism of CNVs, and small CNVs. XHMM w/o WGA detected 855 kb or larger CNVs, including terminal deletion and mosaicism that were undetected by XHMM with WGA. XHMM w/o WGA missed only small CNVs with 220 kb and 221 kb (Table 1). The detection rates of BlueFuse Multi with WGA, XHMM with WGA, and XHMM w/o WGA were 23.8% (5/21), 81.0% (17/21), and 90.5% (19/21), respectively. It was obvious that XHMM is superior to BlueFuse Multi in terms of both the detection limit and the detection rate. The results showed the clear difference between XHMM with WGA and w/o WGA in the terminal deletion case and mosaics. Figure 1 shows the actual data of different D-karyo methods in Case pre-6. BlueFuse Multi could not identify CNVs, and XHMM with WGA showed only one red dot, which was interpreted as normal. As shown in the bar at the bottom of Figure 1, the XHMM without the WGA method successfully detected the 1q terminal deletion with multiple red dots.

### 3.2. Statistical Analysis of WGA vs. w/o WGA

Statistical significance was evaluated with simple linear regression analysis of whether there was any critical difference in performance with or w/o WGA. First, average numbers of aligned reads to the reference genome against total numbers of reads in each case (%filtered mapped reads) were compared (Table 2), leading to detecting the statistical significance between 57.9% in WGA and 77.35% in w/o WGA (*p* < 0.001, 95% confidence interval (CI): 19.31 to 19.59). These data showed that sequenced reads in w/o WGA are efficiently aligned to the reference genome and effectively utilized for the subsequent CNV analysis compared to those in WGA. Next, averages of Z-scores in XHMM on gains and losses, of which regions ≤ 10 Mb were collated, resulting in gains with 3.71 in WGA vs. 4.38 in w/o WGA (*p* < 0.005, 95% CI: 0.251 to 1.100) and losses with −3.48 in WGA vs. −4.10 in w/o WGA (*p* < 0.001, 95% CI: −0.888 to −0.345). This result shows that Z-scores of w/o WGA in XHMM are significantly higher in both gains and losses than those of WGA, as shown in Figure 1, which is assumed to result from less fluctuation in the baseline in w/o WGA compared with WGA.

### 3.3. Submicroscopic Chromosome Abnormalities Detected by D-karyo

**Figure 2:**
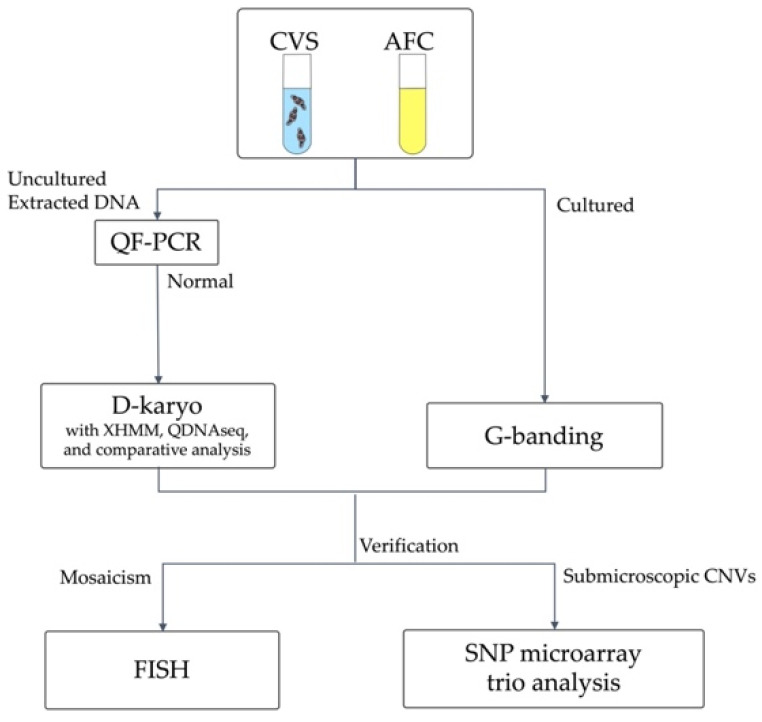
Schematic flowchart of this study. D-karyo was performed on cases excluding aneuploidy for chromosomes 13, 18, 21, and sex chromosomes. The inheritance of cases with submicroscopic CNVs was investigated by trio-based single nucleotide polymerase (SNP) microarray. Abbreviations: CVS, chorionic villus sampling; AFC, amniotic fluid cell; QF-PCR, quantitative fluorescent polymerase chain reaction; CNVs, copy-number variations; XHMM, eXome Hidden Markov Model; QDNAseq, quantitative DNA Sequencing for chromosomal aberrations.

We performed D-karyo analysis on 824 cases (749 CVSs and 75 ACs), as shown in Figure 2. In all cases, G-banding was performed after long-term culture. There were no cases of culture failure. Table 3 summarizes the number and frequency of cases with pathogenic and potential for clinical significance by D-karyo in 824 samples with normal karyotype, according to invasive tests’ main indication. Overall, the positive D-karyo result in all 824 cases was 1.2%. We classified 824 patients by the main indication for invasive tests. The D-karyo positive rate of the AMA group and the NT < 3.5 mm with Down’s screening positive group were as low as 0.7% and 0.8%, respectively. However, the D-karyo positive rates were 2.5% and 5.0%, respectively, in the positive screening group with NT ≥ 3.5 mm and the group in which one or more abnormalities were detected by ultrasound. 

**Figure 3:**
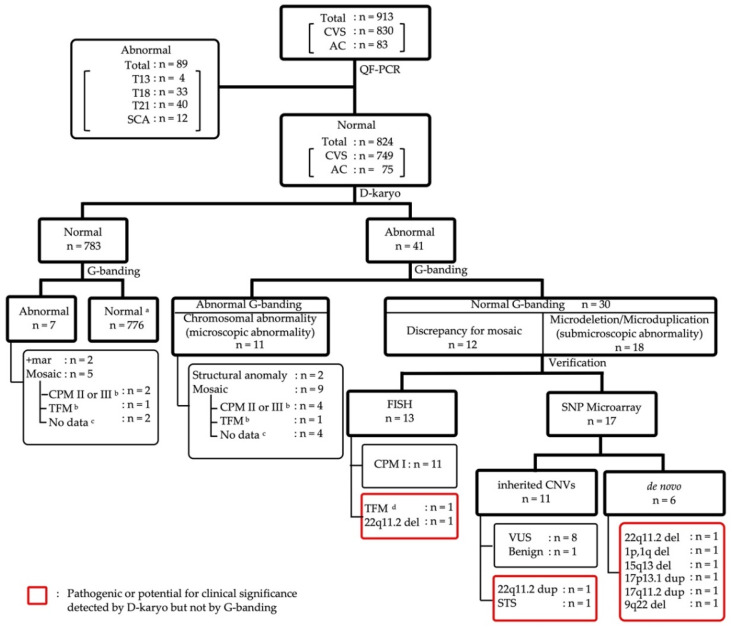
Outcome flowchart for the prospective study with 824 cases based on D-karyo and G-banding. Detailed information on each case was classified based on results of D-karyo and G-banding. Cases in groups surrounded by red squares are indicated pathogenic or potential clinical significance detected by D-karyo but not by G-banding. ^a^, cases which included balanced translocation and chromosomal heteromorphisms. ^b^, cases which were confirmed by chromosome analysis with AC. ^c^, cases that were not able to verify by chromosome analysis with AC. ^d^, cases which were concluded as TFM because outcomes of D-karyo with AC and NIPT were consistent. Abbreviations: CVS, chorionic villus samplings; AC, amniocentesis; SCA, sex chromosome aneuploidy; CPM, confined placental mosaicism; TFM, true fetal mosaicism; STS, steroid sulfatase deficiency; VUS, Variant of Unknown Significance.

Forty-one abnormal cases in D-karyo were subjected to G-banding, resulting in 11 cases with microscopic chromosome aberrations (Case m-1 to Case m-2, and Case mo-1 to Case mo-9) and 18 cases with microdeletions or microduplications (Case s-1 to Case s-18) (Figure 3, Table 4, Table 5 and Table 6).

**Figure 4:**
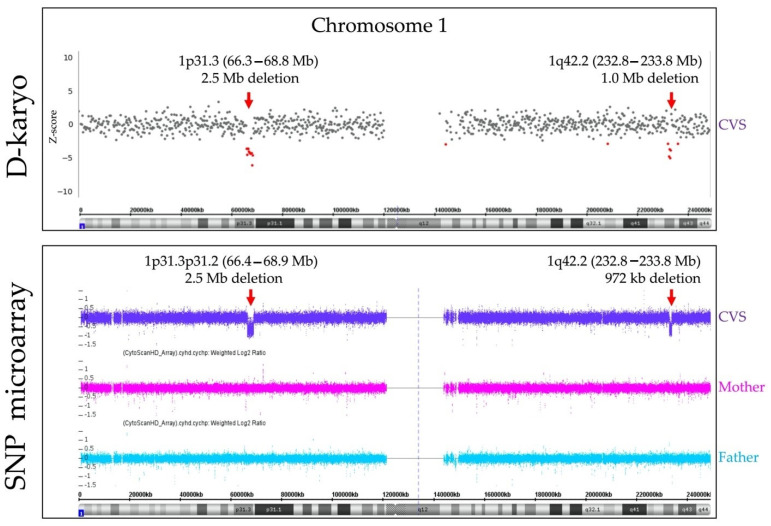
A case for de novo chromosomal inversion with submicroscopic deletions at each breakpoint: Case s-13. (Top) D-karyo outcome. X- and Y-axes represent the physical location in chromosome 1 and Z-score from XHMM, respectively. Points in red indicate regions with Z-score ≤ −2.5. (Bottom) Trio-based SNP microarray verification outcome in Affymetrix CytoScan^TM^ HD. X- and Y-axes represent the physical location in chromosome 1 and Log2 ratio, respectively. The purple, red, and blue arrows represent copy-number alterations in CVS, maternal, and paternal peripheral blood, respectively. Microdeletions of approximately 2.5 Mb and 1.0 Mb (red arrows) were detected by D-karyo and SNP microarray. The outcome of the trio-based SNP microarray indicates that these microdeletions are de novo.

We assessed the pathogenicity in 18 cases of submicroscopic abnormalities (Table 4), according to the ACMG guideline. Two cases with 22q.11.2 deletion syndrome (Case s-12 and Case s-18), a case with 22q.11.2 duplication syndrome (Case s-1), a case with steroid sulfatase (STS) deficiency (Case s-2), and a structural anomaly (Case s-17) were diagnosed as pathogenic, and the other 13 cases resulted in VUS or were benign. In Case s-17 in Table 4, the CVS karyotyping resulted in normal, but CVS D-karyo detected 12.0 Mb deletion of 9q22.2q31.1. Interestingly, the same microdeletion as the D-karyo result was confirmed by the subsequent AC G-banding. The discordance of G-banding results between CVS and AC indicates that it is occasionally more difficult to identify such a small deletion within a broad light band of the chromosome in a CVS specimen than in an AC sample. Although it is commonly assumed that inherited CNVs would not affect phenotypes, there are exceptions in specific heredity patterns. Case s-2 is an example. In this case, we diagnosed STS deficiency caused by Xp22.31 deletion based on the inheritance of hemizygous deletion on the X chromosome. With the exception of a case verified with FISH (Case s-18), CNVs of the other 17 cases were confirmed by trio-based SNP microarray, which showed six de novo CNVs (Case s-12 to Case s-17) and 11 inherited CNVs (Case s-1 to Case s-11) (Figure 3 and Table 4). De novo pericentric inversion of chromosome 1 was detected in Case s-13, which caused both 2.5 Mb deletion in the short arm and 1.0 Mb deletion in the long arm of chromosome 1 (Figure 4 and Table 4).

### 3.4. Microscopic Chromosome Anomalies

Case m-1 was strongly suspected of having structural abnormality of chromosome 18 by QF-PCR, and G-banding subsequently confirmed the aberration. An additional segment on the 11q terminal in Case m-2 was observed as 15.5 Mb duplication of 19q13.31 to the terminal segment detected by D-karyo, which was verified as de novo by trio-based karyotyping.

Low-frequency mosaicism (10–20%) in Case mo-1 to Case mo-4 was detected by D-karyo and G-banding with CVS samples. The type of CPM was II or III when G-banding was normal in AC (Table 6). By comparison, Case mo-5 with high-frequency mosaicism (80%) in D-karyo and full trisomy eight in G-band with CVS shows mosaicism in G-band with AC, indicating TFM.

### 3.5. Discrepancy between D-karyo and G-banding

Eleven cases (Case mo-10 to Case mo-20) in Table 6 are mosaic cases with 10–80% of segmental mosaicism, which was detected in D-karyo and FISH, but not detected in G-banding of CVS. All of these cases showed the normal AC G-banding results. Therefore, they were diagnosed as CPM type I. Although AC was not available in Cases mo-18, mo-19, and mo-20, they were classified as CPM type I according to mosaicism’s relative frequency [29]. In one case (Case mo-21) initially suspected as trisomy 16 by NIPT, D-karyo revealed mosaic trisomy 16 (20%), confirmed by uncultured FISH. We concluded this case as TFM, although AC G-banding resulted in normal (Table 6).

### 3.6. Limitation of D-karyo

As expected, it appears difficult for D-karyo to identify structural chromosome anomalies with no CNV alterations, such as balanced reciprocal translocations and heteromorphism. As shown in Table 7, two out of seven false negative cases were small supernumerary marker chromosome (sSMC), which cannot be detected even by SNP microarray. The reason why sSMC could not be detected by D-karyo is a shortage of sufficient reads due to less unique sequences, for instance, in Case fn-1 and Case fn-2 (Table 7). Trio-based G-banding and FISH revealed that Case fn-2 showed 47, XY,+psu idic(15)(q11)pat, which was assumed to be a benign sSMC not representing any disease-associated phenotype.

The remaining five cases are chromosomal aneuploidy mosaics. In Case fn-3 to Case fn-7, although CVS D-karyo resulted in normal, G-banding detected mosaicism, confirmed by uncultured FISH as low-frequency mosaicism (12–30%). Case fn-3 and Case fn-4 were considered CPM type II or III, and Case fn-5 was deemed TFM (Table 7). In mosaic cases, the G-band results may not be accurate due to changes in the mosaic rate during long-term culture. It can be also explained that the result of D-karyo and G-band may be different in the mosaic cases, because the mosaic ratio differs depending on the tissue origin.

## 4. Discussion

Conventional cytogenetic G-banding of prenatal specimens has the disadvantages of a long duration of cell culture, lower resolution than postnatal specimen testing, changing mosaic frequency during culture, and examiner dependency. Many obstetricians have experienced the fetus with a normal G-band result, which was found to be microdeletion syndrome after birth. Thus, submicroscopic CNVs are overlooked by G-banding. CMAs, such as SNP microarray, would replace G-banding. Although CMA is a useful method for accurately diagnosing submicroscopic CNVs and low frequent mosaicism, it is expensive, and has a low throughput and a long turnaround time (TAT).

In this study, we introduced a new prenatal screening method named D-karyo. D-karyo utilizes the platform of PGT-A and is cost-effective and time-saving. The original PGT-A protocol was not useful for a prenatal CNV screening because the resolution of PGT-A may be little better than or similar to that of the G-banding. It was thus required to increase the CNVs detection rate and decrease the detection limit. As shown in this study, three steps were used to achieve a high detection rate. The first step was the introduction of XHMM to detect CNVs. The second was addressing the challenge of the approach w/o WGA. In the field of assisted reproductive technologies and preimplantation genetic testing, WGA is necessary because of the small amount of gDNA obtained from fertilized egg cells. However, in prenatal clinical practice, we can obtain sufficient DNA from chorionic villi or amniotic fluid cells. Therefore, we proposed the option w/o WGA. We verified whether the CNV detection rate could improve by omitting WGA. The third step was to introduce two further analytical tools in addition to XHMM for increased mosaic detection.

In the initial validation study, we first confirmed that XHMM could detect significantly smaller CNVs than BlueFuse Multi (the original PGT-A method). Next, we compared D-karyo’s detection rate of CNVs with and w/o WGA using already known pathogenic CNV samples. We obtained a higher detection rate and smaller CNVs as a detection limit in the XHMM w/o WGA than with WGA. In one case, D-karyo detected terminal microdeletion with 2.2 Mb in a sample w/o WGA, while it was not depicted in an amplified specimen by WGA. It is assumed that it would be difficult to obtain enough reads for evaluation in the WGA sample because the microdeletion was located on the chromosomal terminal segment with less-unique repetitive sequences.

In specimens with WGA, XHMM showed a higher and more stable detection rate than BlueFuse Multi Software. BlueFuse Multi, used in a routine PGT-A procedure, was not available to be used w/o WGA because background noise interrupted accurate detection of CNVs due to incorrect normalization. Overall, based on these results, it was suggested that the VeriSeq system w/o WGA would be most suitable for CNV analysis on prenatal samples to detect submicroscopic CNVs. The result of the statistical analysis of WGA vs. w/o WGA showed that Z-scores of XHMM w/o WGA were significantly higher in both gains and losses than those of WGA, which may be because of the lower fluctuation on the baseline in the samples w/o WGA compared to the specimens with WGA. These statistical analyses suggest that the VeriSeq system w/o WGA would enable the detection of much smaller submicroscopic CNVs more efficiently and effectively than with WGA to screen prenatal chromosomal aberrations. In our case series, submicroscopic chromosome abnormalities ≥1 Mb were completely detected by D-karyo. This is notable because most microdeletion/microduplication syndromes are based on 1–3 Mb CNVs. Furthermore, for the clinical trial study, to investigate CNVs w/o WGA, we added QDNAseq to address full mosaicism, and as a comparative analytical tool to XHMM to detect low-frequency mosaic. D-karyo would be a useful tool to detect the low frequency of mosaicism and could offer clues for structural anomalies with CNV alterations and derivation of an additional segment. Our study results also suggest that it would be helpful to use D-karyo in addition to G-banding, to provide more accuracy and prevent overlooking of small disease-associated CNVs and low-frequency mosaicism. Regarding mosaicism, we should carefully consider how much or which part of the sample is included in the source material, especially in CVS, because the location or quantity of CVS may influence the mosaic frequency. Structural chromosome abnormalities without CNV alterations clearly cannot be detected by D-karyo or even CMA. D-karyo also has a limitation in detecting the low and unstable frequency of mosaicism.

In the clinical trial, D-karyo detected 41 abnormalities (5.0%) out of 824 cases, including 11 microscopic and 18 submicroscopic abnormalities, and 12 discrepancy cases with mosaic. Of these, 30 (3.6%) abnormalities were not observed by G-banding. In 30 cases of positive D-karyo and negative G-banding, trio-based SNP microarray or FISH were performed to confirm pathogenicity. As a result, ten cases (1.2%) were finally categorized as disease-associated chromosome anomalies and CNVs, as shown in Figure 3. According to the main indication for invasive tests, the frequency of the pathogenic and the potential for clinical significance by D-karyo with normal karyotype, the D-karyo positive rate of the AMA group, and the NT < 3.5 mm with Down’s screening positive group were as low as 0.7% and 0.8%, respectively. However, the D-karyo positive rates were 2.5% and 5.0%, respectively, in the positive screening group with NT ≥ 3.5 mm and the group in which one or more abnormalities were detected by ultrasound. Wapner et al. [3] reported that the frequency of pathogenic and potential for clinical significance CMA in the AMA group and positive Down screening group was 1.7% and 1.6%, respectively, and the rate in anomaly on ultrasonography group was 6.0%. Xie et al. [32] also reported that pathogenic CNVs were detected in 4.76% of the ultrasound structural malformation group. Therefore, our D-karyo study results were considered to be close to the reported CMA results.

**Figure 5:**
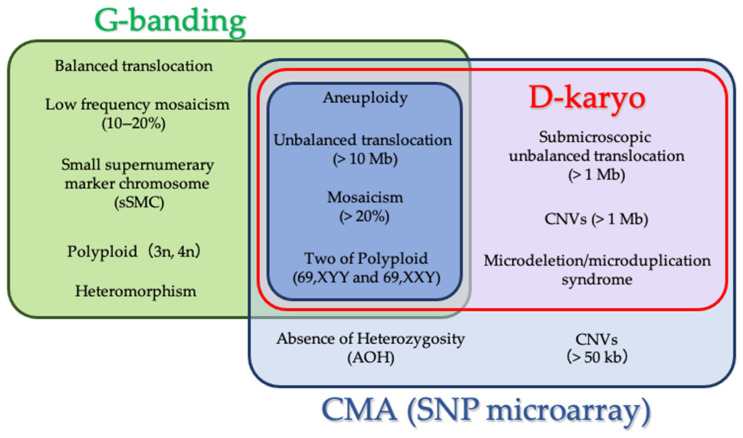
Venn diagram of D-karyo, G-banding, and CMA. The benefits and limitations of each technology are demonstrated based on this study.

For prenatal diagnosis based on genetic analysis, it is necessary to overcome common challenges to achieve accurate assessment in the clinical setting, focusing on three points: (i) the detectable size of CNV by D-karyo; (ii) the detection sensitivity; and (iii) the detection rate of mosaicism. The benefits and limitations of G-banding, CMA, and D-karyo are shown in Figure 5.

(i) A previous report described that the minimum size of CNV detected by the VeriSeq system was approximately 10 Mb [25]. The detected minimum CNV size in this study using D-karyo was 855 kb for deletion in the preliminary experiment w/o WGA, and 555 kb for deletion in the prospective study of 824 cases, suggesting that D-karyo can detect CNVs of less than 1.0 Mb.

(ii) Detection sensitivity was assessed on phenotypic anomaly causative CNVs associated with CNV syndrome in DECIPHER, and pathogenic CNVs regions in ClinGen. Thirteen cases of chromosome anomalies, including submicroscopic abnormalities such as CNV syndrome and microscopic abnormalities, including Emanuel syndrome, were detected both in the preliminary and prospective studies. To increase the test’s accuracy, the information of D-karyo helped examine G-banding, e.g., Karyotype of Case s-13 was concluded as inv(1)(q31q42) by referring to CNVs on 1p31.3 and 1q42.2, which were detected by D-karyo before G-banding. Furthermore, detecting such small deletions associated with chromosomal inversion suggests that it is crucial to identify submicroscopic CNVs using sufficient caution in balanced rearrangement [33]. In our preliminary experiment, Case pre-17, 8.9 Mb duplication and 8.8 Mb deletion in the terminal region of 11q were detected in D-karyo, whereas it was difficult to verify in G-banding. In Case s-17, deletion within the light band in G-banding was not observed because of the limitation of the resolution for CVS. Nonetheless, the deletion was detected in D-karyo and finally confirmed in AC. The demand for detecting submicroscopic chromosomal abnormalities has recently increased. In 1991, Warburton et al. reported that phenotypic abnormalities were found in 6–7% of de novo balanced translocations [34]. Subsequently, several reports were published on submicroscopic pathogenic CNVs in cases thought to be benign. It was reported that CMA was performed on phenotypic abnormal children with diagnoses of equilibrium chromosomal translocation and, in fact, submicroscopic CNVs were detected in about 40% of cases [35,36,37]. Furthermore, a recent long-term follow-up study of de novo balanced chromosomal rearrangement detected prenatally reported that morbidity risk was upwardly revised to 27%, after being initially reported as 6–7% 30 years before; sequencing of the breakpoint was also recommended [33]. These observations indicate that the importance of investigating CNVs associated with de novo balanced translocation to address the long-term clinical significance appears to be gradually increasing, as shown here. In prenatal diagnosis, it is clearly required to detect submicroscopic CNVs at breakpoints in cases with de novo balanced translocation. As previously reported [1,2], the resolution in prenatal specimens is lower than that in postnatal specimens. This was also true in our study, in which CVS specimens may have a lower resolution than amniotic fluid specimens. To raise the level of prenatal chromosomal diagnosis to the level of postnatal diagnosis, the D-karyo approach proposed in this study is considered to be one of the breakthroughs.

(iii) There have been many validation studies on PGT-A using the VeriSeq system for trophectoderm. According to previous studies, it is expected to detect mosaic frequency over 20% [38,39]. We confirmed the detection rate of more than 20% of mosaicism in D-karyo. This detection rate is comparable to that of CMA, indicating that D-karyo would help detect mosaicism. For example, Case mo-21, which was suspected trisomy 16 in NIPT, showed normal G-banding with AC, which initially led to CPM type I. However, the outcome in D-karyo showed approximately 20% of mosaic trisomy 16, which finally resulted in the assessment as TFM. It is conventionally crucial to verify CPM or TFM with AC, of which G-banding does not necessarily reflect the accurate mosaic rate due to the accompanying culturing process. The discrepancy of mosaicism rates before and after cell culture often arises as a disadvantage because proliferation speeds with and without chromosomal abnormality are inconsistent. Therefore, this study indicates that the technical process of D-karyo in which gDNA is extracted directly from uncultured materials would make it possible to avoid such a culturing bias and lead to the detection of a significantly more accurate frequency of mosaicism.

Finally, regarding the advantages of using D-karyo, this approach has several benefits compared to CMA, as shown in Table 8. As a first benefit, SNP microarray using CytoScan^TM^ HD or 750K takes 3–4 days for a trio of samples, whereas D-karyo w/o WGA finishes within 10.5 h for 24 samples. Another advantage of D-karyo w/o WGA is that it requires an amount of gDNA as small as 1 ng, compared with 250 ng required for SNP microarray. Based on these facts, we believe that using D-karyo combined with conventional prenatal testing helps save time, cost, and DNA of a specimen.

## 5. Conclusions

D-karyo proposed in this paper, applying a high throughput PGT-A methodology, can detect submicroscopic CNVs in a short TAT at a low cost. We showed in this study that D-karyo can be used as a rapid screening method to compensate for the weaknesses of G-banding. Rapid screening for CNVs ≥ 885 kb on all chromosomes, and assessment of microdeletion/microduplication syndrome in just 10.5 h, appears to be a significant technological advance.

We do not consider D-karyo to be a replacement for CMA. We position D-karyo as a rapid screening test, and trio-based CMA as a definitive test to confirm the actual size and inheritance of CNVs. We consider the clinical flow of performing trio CMA when D-karyo test results are positive. The efficient use of the D-karyo rapid screening test in combination with G-banding could increase the detection rate of chromosomal abnormalities, reduce unnecessary CMA tests, and increase the accuracy of prenatal genetic testing.

A future task is to increase the D-karyo accuracy of detecting CNVs of less than 1 Mb.

## Figures and Tables

**Table 1:** Comparison of D-karyo detection of copy-number variations (CNVs) in cases with known pathogenic CNVs.

Preliminary Experiment Case	SNP Microarray	Deletion or Duplication	Syndrome	Size	D-karyo Method		
					BlueFuse Multi (Original PGT-A Method)	XHMM,	XHMM,
					with WGA	with WGA	w/o WGA
pre-1	arr[hg19] 1q21.1q21.2(145,895,746–147,995,251) × 3	1q interstitial duplication		2.1 Mb	-	+	+
pre-2	arr[hg19] 2p13.3p12(70,817,760–76,194,406) × 1	2p interstitial deletion		5.3 Mb	+	+	+
pre-3	arr[hg19] 4p16.3p15.33(68,345–14,582,038) × 1	4p terminal deletion	Wolf-Hirschhorn Syndrome	14.5 Mb	+	+	+
pre-4	arr[hg19] 13q33.1q34(101,714,084–115,107,733) × 1	13q terminal deletion		13.4 Mb	+	+	+
pre-5	arr[hg19] 5p13.2(34,507,645–36,691,250) × 1	5p interstitial deletion		3.4 Mb	-	+	+
pre-6	arr[hg19] 1q44(247,001,499–249,224,684) × 1	1q terminal deletion		2.2 Mb	-	-	+
pre-7	arr[hg19] (X)x3,17p11.2(16,763,697–20,463,423) × 1	17p interstitial deletion	Smith-Magenis Syndrome	3.7 Mb	-	+	+
pre-8	arr[hg19] 11q23.3q25(116,683,754–134,937,416) × 3,	11q terminal duplication	Emanuel Syndrome	18.3 Mb	+	+	+
	22q11.1q11.21(16,888,899–20,312,661) × 3	22q interstitial duplication		3.4 Mb	-	+	+
pre-9	arr[hg19] 1q24.2q24.3(169,549,903–172,184,068) × 1	1q interstitial deletion		2.6 Mb	-	+	+
pre-10	arr[hg19] 10q26.2q26.3(128,251,907–135,427,143) × 1,	10q terminal deletion		7.2 Mb	+	+	+
	17q25.1q25.3(73,011,284–81,041,938) × 3	17q terminal duplication		8.0 Mb	+	+	+
pre-11	arr[hg19] 7q11.22(69,755,328–69,976,338) × 1	7q interstitial deletion		221 kb	-	-	-
pre-12	arr[hg19] 19p12(20,696,177–23,131,879) × 3	19p interstitial duplication		2.4 Mb	-	+	+
pre-13	arr[hg19] 1p36.33p36.31(849,466–7,027,594) × 1,	1p terminal deletion	1p36 deletion Syndrome	6.2 Mb	+	+	+
	1p36.23p36.22(7,391,956–10,686,850) × 2–3	1p interstitial duplication (50% mosaic)		3.3 Mb	-	-	+
pre-14	arr[hg19] 17p13.3(1,061,401–1,281,546) × 3 mat	17p interstitial duplication		220 kb	-	-	-
pre-15	arr[hg19] 22q11.21(18,648,855–21,800,471) × 1	22q interstitial deletion	22q11.2 deletion Syndrome	3.2 Mb	-	+	+
pre-16	arr[hg19] 7q11.23(72,611,954–74,245,940) × 1 dn	7q interstitial deletion	Williams Syndrome	1.6 Mb	-	+^a^	+^a^
pre-17	arr[hg19] 11q21q22.1(93,535,045–97,323,000) × 3,	11q interstitial duplication	Jacobsen syndrome	8.9 Mb	+	+	+
	11q22.1q24.2(97,268,927–126,080,195) × 2–3,	11q interstitial duplication (50% mosaic)		28.8 Mb	+	+	+
	11q24.2q25(126,048,147–134,937,416) × 1	11q terminal deletion		8.8 Mb	+	+	+
pre-18	arr[hg19] 1p36.33p36.32(849,466–3,763,567) × 1,	1p terminal deletion	1p36 deletion Syndrome	2.9 Mb	-	+	+
	5q35.1q35.3(172,600,252–180,715,096) × 3	5q terminal duplication		8.1 Mb	+	+	+
pre-19	arr[hg19] Xq22.1q22.2(102,273,407–103,223,398) × 2	Xq interstitial duplication	Pellzeus-Merzbacher Disease	950 kb	-	+	+
pre-20	arr[hg19] Xp22.33(168,551–1,023,657) × 1	Xp terminal deletion	Leri-Weill dyschondrosteosis	855 kb	-	+^a^	+^a^
pre-21	arr[hg19] 5q35.2q35.3(175,469,493–177,439,550) × 1	5q interstitial deletion	Sotos Syndrome	1.97 Mb	-	+	+
			Detection limit		≥5.3 Mb	>2.2 Mb	≥855 kb
			Detection rate		23.8%	81.0%	90.5%

+, CNVs were detected by both XHMM automatic detection system and the additional standard for CNV detection: three consecutive target regions with Z-score of 2.5 or more. -, CNVs were undetected. +^a^, CNVs were detected by only the additional standard for CNV detection.

**Table 2:** Statistical difference between D-karyo with WGA and w/o WGA.

Examination Result	with WGA Average, (n)	w/o WGA Average, (n)	*p*-Value (95% Cl)
%Filtered Mapped Reads	57.9 (357)	77.35 (495)	0.001 (19.31 to 19.59)
Z-score for CNVs Loss	−3.48 (6)	−4.10 (11)	0.001 (−0888 to −0.345)
Z-score for CNVs Gain	3.71 (19)	4.38 (25)	0.005 (0.251 to 1.100)

%filtered mapped reads; average numbers of aligned reads to the reference genome against total numbers of reads in each case; Z-score for CNVs Loss or Gain; averages for Z-score calculated by XHMM software. 95% CI, 95% confidence interval.

**Table 3:** The number and frequency of cases with pathogenic and potential for clinical significance by D-karyo in 824 samples with normal karyotype, according to the main indication for invasive tests.

Main Indication for Invasive Tests	Number	Pathogenic Number (%)		Potential for Clinical Significance Number (%)		Pathogenic + Potential for Clinical Significance Number (%)	
Any	824	6	(0.7)	4	(0.5)	10	(1.2)
Advanced maternal age *	494	1	(0.2)	3	(0.6)	4	(0.8)
Positive Down’s screening test							
NT < 3.5 mm	149	1	(0.7)	0	(0.0)	1	(0.7)
NT ≥ 3.5 mm	79	2	(2.5)	0	(0.0)	2	(2.5)
One or more structural anomalies on ultrasonography	40	2	(5.0)	0	(0.0)	2	(5.0)
Other **	62	0	(0.0)	1	(1.6)	1	(1.6)

* Maternal age ≥ 35 years old; ** Other indications include family history, previous pregnancy with chromosomal abnormalities, and parental anxiety. NT, nuchal translucency.

**Table 4:** Cases of submicroscopic abnormalities (s) detected by D-karyo.

Case No.	D-karyo	D-karyo Size	FISH or CMA (SNP Microarray)	CMA Size	ACMG Score	Final Assessment
s-1	22q11.21 (18.5–22.0 Mb)	3.5 Mb dup	arr[hg19] 22q11.21(18,916,842–21,464,764) × 3 pat	2.5 Mb dup	Pathogenic (1)	inherited CNVs, 22q11.2 duplication syndrome
s-2	Xp22.31 (6.4–8.2 Mb)	1.8 Mb del	arr[hg19] Xp22.31(6,455,151–8,135,568) × 0 mat	1.7 Mb del	Pathogenic (1)	inherited CNVs, Steroid sulfatase deficiency
s-3	10p13 (15.9–17.2 Mb)	1.3 Mb del	arr[hg19] 10p13(15,966,079–17,261,220) × 1 mat	1.3 Mb del	VUS (−0.15)	inherited CNVs, VUS
s-4	18q21.32 (56.3–58.9 Mb)	2.6 Mb del	arr[hg19] 18q21.32(56,476,158–58,168,301) × 1 pat	1.7 Mb del	VUS (−0.15)	inherited CNVs, VUS
s-5	2p25.3 (0.3–0.9 Mb)	600 kb del	arr[hg19] 2p25.3(343,157–898,179) × 1 mat	555 kb del	VUS (−0.15)	inherited CNVs, VUS
s-6	2q11.1q11.2 (96.5–98.4 Mb)	1.9 Mb del	arr[hg19] 2q11.1q11.2(96,747,802–98,141,300) × 1 mat	1.4 Mb del	VUS (−0.15)	inherited CNVs, VUS
s-7	1p31.1 (76.3–77.9 Mb)	1.6 Mb del	arr[hg19] 1p31.1(76,444,107–77,616,384) × 1 mat	1.2 Mb del	VUS (−0.15)	inherited CNVs, VUS
s-8	10q11.22q11.23 (46.1–51.5 Mb)	5.4 Mb del	arr[hg19] 10q11.22q11.23(46,153,831–51,903,756) × 1 pat	5.8 Mb del	VUS (0.75)	inherited CNVs, VUS
s-9	Yq11.221 (16.3–18.0 Mb)	1.7 Mb dup	arr[hg19] Yq11.221(17,041,429–17,668,698) × 4 pat	627 kb dup	VUS (−0.75)	inherited CNVs, VUS
s-10	Xp22.31 (6.2–8.2 Mb)	2.0 Mb dup	arr[hg19] Xp22.31(6,455,151–8,143,509) × 3 pat	1.7 Mb dup	VUS (−0.15)	inherited CNVs, VUS
s-11	Xp22.31 (6.5–8.2 Mb)	1.7 Mb trip	arr[hg19] Xp22.31(6,455,151–8,135,644) × 3 mat	1.7Mb trip	Benign (−1.05)	inherited CNVs, Benign
s-12	22q11.21 (18.5–22.0 Mb)	3.5 Mb del	arr[hg19] 22q11.21(18,648,855–21,800,471) × 1 dn	3.2 Mb del	Pathogenic (1)	de novo, 22q11.2 deletion syndrome
s-13	1p31.3 (66.3–68.8 Mb)	2.5 Mb del	arr[hg19] 1p31.3p31.2(66,456,464–68,991,911) × 1 dn,	2.5 Mb del	VUS (0.15)	de novo, VUS, karyotype
	1q42.2 (232.8–233.8 Mb)	1.0 Mb del	1q42.2(232,844,622–233,817,186) × 1 dn	972 kb del	VUS (0.15)	46,XX,inv(1)(p31q42)dn,inv(9)(p12q13)mat
s-14	15q13.1q13.3 (28.6–31.3 Mb)	2.7 Mb del	arr[hg19] 15q13.1q13.2(29,069,000–30,370,018) × 1 dn	1.3 Mb del	VUS (0.15)	de novo, VUS
s-15	17p13.1 (8.0–10.0 Mb)	2.0 Mb dup	arr[hg19] 17p13.1(8,004,278–9,824,210) × 3 dn	1.8 Mb dup	VUS (0.15)	de novo, VUS
s-16	17q11.2 (28.7–30.2 Mb)	1.5 Mb dup	arr[hg19] 17q11.2(29,000,429–30,409,336) × 3 dn	1.4 Mb dup	VUS (0.45)	de novo, VUS
s-17	9q22.2q31.1 (12.0 Mb del)	12.0 Mb del	arr[hg19] 9q22.2q31.1(92,245,335–104,251,390) × 1 dn	12.0 Mb del	Pathogenic (1)	de novo CNVs, Pathogenic
s-18	22q11.2 (18.6–21.5 Mb)	2.9 Mb del	ish(TUPLE1 × 1)[20/20]		Pathogenic (1)	unknown, 22q11.2 deletion syndrome

Abbreviation: VUS, variant of unknown significance. del, deletion; dup, duplication; trip, triplication.

**Table 5:** Cases of microscopic abnormalities (m) detected by D-karyo.

Case No.	Type	D-karyo	karyotype	FISH or CMA (SNP Microarray)	karyotype (AC)	Final Assessment
m-1	CVS	18p monosomy, 18q trisomy	46,XY,i(18)(q10)		-	Match with karyotype, Structural anomaly
m-2	CVS	19q13.31qter (43.6–59.1 Mb)	46,XX,der(11)t(11;19)(q25;q13.3)dn		-	Match with karyotype, Structural anomaly

Abbreviation: CVS, chorionic villus samplings; AC, amniocentesis; FISH, fluorescent in situ hybridization; CMA, chromosomal microarray analysis.

**Table 6:** Cases with mosaicism (mo) in D-karyo.

Case No.	Type	D-karyo	karyotype	FISH or CMA (SNP Microarray)	karyotype (AC)	Final Assessment
mo-1	CVS	mosaic trisomy 2 (20%)	47,XY,+2[6]/46,XY[44]	nuc ish(MAL×3)[2/10]	46,XY	CPM type II or III
mo-2	CVS	mosaic trisomy 6 (10%)	47,XX,+6[5]/46,XX[45]	nuc ish(SOD2×3)[3/50]/(SOD2×1)[2/50]	46,XX	CPM type II or III
mo-3	CVS	mosaic trisomy 2 (10%)	47,XX,+2[2]/46,XX[28]	nuc ish(MAL×3)[5/50]	46,XX	CPM type II or III
mo-4	CVS	mosaic trisomy 2 (20%)	47,XY,+2[5]/46,XY[45]	nuc ish(MAL×3)[10/50]	46,XY	CPM type II or III
mo-5	CVS	mosaic trisomy 8 (80%)	47,XY,+8	nuc ish(CEBPD×3)[25/30]	47,XY,+8[14]/46,XY[36]	TFM
mo-6	CVS	mosaic monosomy X (80%)	45,X[8]/46,XX[42]	nuc ish(DXZ1×1)[26/50]	-	Mosaic, no data
mo-7	CVS	mosaic monosomy X (30%)	45,X[18]/46,XX[32]	nuc ish(DXZ1×1)[14/50]	-	Mosaic, no data
mo-8	CVS	mosaic monosomy X (80%)	47,XXX[46]/45,X[4]	nuc ish(DXZ1×1)[31/50]/(DXZ1×3)[17/50]	-	Mosaic, no data
mo-9	CVS	mosaic monosomy X (10–20%)	45,X[5]/46,XX[45]	nuc ish(DXZ1×1)[4/50]	-	Mosaic, no data
mo-10	CVS	mosaic trisomy 7 (10–20%)	46,XY	nuc ish(D7S741×3)[4/50]	46,XY	Discrepancy, CPM type I
mo-11	CVS	mosaic trisomy 20 (20–25%)	46,XY	nuc ish(CST7×2)[50]	46,XY	Discrepancy, CPM type I
mo-12	CVS	partial mosaic trisomy 8 (60–80%) 8q23.3q24.3(114.1 Mb dup)	46,XY	nuc ish(MYC×3)[3/50]	46,XY	Discrepancy, CPM type I
mo-13	CVS	mosaic trisomy 7 (10–20%)	46,XY	nuc ish(D7S741×2)[50]	46,XY	Discrepancy, CPM type I
mo-14	CVS	mosaic trisomy 7 (40%)	46,XY	nuc ish(D7S741×2)[50]	46,XY	Discrepancy, CPM type I
mo-15	CVS	mosaic monosomy 21 (10–20%)	46,XX	nuc ish(D21S342×1)[2/200]	-	Discrepancy, CPM type I
mo-16	CVS	mosaic trisomy T13 (10–20%)	46,XX,inv(9)(p12q13)	nuc ish(RB1×3)[2/50]	46,XX,inv(9)(p12q13)	Discrepancy, CPM type I
mo-17	CVS	partial mosaic trisomy 8 (30–40%) 8p21.2q24.3(26.3–140.4)x3	46,XY	nuc ish(CEBPD×3,MYC×3)[5/50]	46,XY	Discrepancy, CPM type I
mo-18	CVS	mosaic monosomy X (10–20%)	46,XY	nuc ish(DXZ1×1,DYZ3×1)[50/50]	-	Discrepancy, CPM type I
mo-19	CVS	mosaic monosomy X (10–20%)	46,XY	nuc ish(DXZ1×1)[1/50]	-	Discrepancy, CPM type I
mo-20	CVS	mosaic trisomy 7 (20%)	46,XX	nuc ish(D7S741×3)[2/100]	-	Discrepancy, CPM type I
mo-21	AC	mosaic trisomy 16 (20%)	46,XX	nuc ish(SHCBP1×3)[3/100]	-	Discrepancy, TFM, NIPT T16 positive

Karyotyping of AC was performed to verify abnormalities detected in CVS. Abbreviation: CVS, chorionic villus samplings; AC, amniocentesis; FISH, fluorescent in situ hybridization; CMA, chromosomal microarray analysis; CPM, confined placental mosaicism; TFM, true fetal mosaicism.

**Table 7:** Cases which showed a false negative (fn) in D-karyo.

Case No.	Sample Type	D-karyo	karyotype	FISH	karyotype (AC)	Final Assessment
fn-1	CVS	normal XY	47,XY,+mar		-	+mar (bisatellited sSMC)
fn-2	CVS	normal XY	47,XY,+psu idic(15)(q11)pat	ish psu idic(15)(q11)(D15Z1++,SNRPN-)	-	+mar (bisatellited sSMC)
fn-3	CVS	normal XX	47,XX,+2[10]/46,XX[70]	nuc ish(MAL×3)[6/50]	46,XX	CPM type II or III
fn-4	CVS	normal XX	47,XX,+8[36]/46,XX[14]	nuc ish(CEBPD×3)[13/50]	46,XX	CPM type II or III
fn-5	AC	normal XX	47,XX,+13[7]/46,XX[43]	nuc ish(RB1×3)[7/50]		TFM, NIPT T13 positive
fn-6	CVS	normal XX	45,X[12]/46,XX[88]	nuc ish(DXZ1×1)[10/100]	-	SCA mosaicism
fn-7	CVS	normal XX	47,XXX[9]/46,XX[41]	nuc ish(DXZ1×3)[15/50]	-	SCA mosaicism

Karyotype (AC) represents outcomes in AC that were performed for the verification of abnormalities found in CVS. Abbreviation: CVS, chorionic villus samplings; AC, amniocentesis; FISH, fluorescent in situ hybridization; CPM, confined placental mosaicism; TFM, true fetal mosaicism, sSMC, small supernumerary marker chromosome.

**Table 8:** Characteristics of CMA and D-karyo.

Evaluation Item	D-karyo	CMA (SNP Microarray)
Application	Screening test	Diagnostic test
Platform	VeriSeq PGS kit	CytoScan HD or 750K array
Amount of gDNA	1 ng	250 ng
WGA	-	-
Sample number/assay	24	4
Analyzing time	10.5 h	3–4 days
Analytic software	XHMM (CNV) QDNAseq (mosaicism) Comparative analysis (mosaicism)	Chromosome Analysis Suite
Resolution	>1 Mb	>50 kb

## Data Availability

The datasets presented in this study are available from the corresponding author. The data are not publicly available due to the individual private information.
